# Sauna-Induced Body Mass Loss in Young Sedentary Women and Men

**DOI:** 10.1155/2014/307421

**Published:** 2014-12-31

**Authors:** Robert Podstawski, Tomasz Boraczyński, Michał Boraczyński, Dariusz Choszcz, Stefan Mańkowski, Piotr Markowski

**Affiliations:** ^1^Department of Physical Education and Sport, University of Warmia and Mazury in Olsztyn, Prawocheńskiego 7, 10-720 Olsztyn, Poland; ^2^Jozef Rusiecki Olsztyn University College, Bydgoska 33, 10-243 Olsztyn, Poland; ^3^Department of Heavy Duty Machines and Research Methodology, Faculty of Technical Sciences, University of Warmia and Mazury in Olsztyn, Oczapowskiego 11, 10-719 Olsztyn, Poland

## Abstract

The aim of the study was to evaluate the relationship between body mass index (BMI) and body mass loss (BML) induced by thermal stress in a dry sauna. The study was conducted on a group of 674 sedentary students, 326 women and 348 men aged 19-20. The correlations between BMI scores and BML were determined. The subjects were placed in supine position in a dry sauna for two sessions of 10 minutes each with a 5-minute break. The influence of BMI on the amount of BML in the sauna was determined by nonlinear stepwise regression. The smallest BML was noted in underweight subjects; students with normal weight lost more weight, whereas the greatest BML was reported in overweight and obese subjects. Persons with a high BMI are at higher risk of dehydration, and they should pay particular attention to replenishing fluids during a visit to the sauna. The proposed equations for calculating BML based on a person's BMI can be useful in estimating the amount of fluids that should be replenished by both men and women during a visit to a dry sauna.

## 1. Introduction

The popularity of sauna bathing, a traditional activity known since the ancient times, has been revived in the past decades in Europe and around the world. At present, dry sauna is widely used in sport, recreation, and rehabilitation. Researchers are of the opinion that sauna does not pose a risk for healthy individuals, including children and elderly people [1, 2]. Sauna bathing significantly influences the function of many bodily organs and systems [3–5]. When used appropriately, sauna has a positive effect on skeletal muscles by speeding up the excretion of metabolic waste [1]. Sauna baths are most often practiced for recovery after exercise [6, 7]. A single visit to a Finnish sauna (three sessions of 10 min each at 90°C and 10% relative air humidity) directly after 30 minutes of aerobic exercise reduces oxidative stress [8]. Heat increases the effectiveness of muscle restitution processes; it improves the flexibility and extensibility of connective tissue structures, which increases overall flexibility levels [9]. Sauna is used in sports to maximize the athletes' physical and psychological recovery [10, 11]. Despite the above, uncontrolled sauna bathing could pose a risk of various disorders, such as severe dehydration, heat exhaustion, stroke, and burn [12–14].

Dry sauna bathing influences mainly physical thermoregulation of the human body, and hot air in the sauna affects mostly the skin and cardiovascular and respiratory systems [13]. The first symptom of sauna bathing is an increase in skin temperature to around 40°C and in body temperature from 38°C to 39°C, which contributes to an imbalance between heat production and evacuation [15]. The specific heat of bodily tissues (the amount of energy required to increase the temperature of 1 kg of bodily tissue by 1°C) approximates 3.5 kJ/°C/kg [16]. The body has a biphasic response to thermal stress. In the first phase, excess heat is evacuated by vasodilation and increased fluid excretion through the sweat glands [1]. Persistent thermal stress leads to a gradual increase in bodily temperature (phase two), accompanied by a shift of the acid-base balance towards acidosis, increased blood pressure, higher heart, and breathing rate [17]. Prolonged heat stress promotes the loss of minerals, including sodium, potassium, magnesium, and iron, as well as ammonia and urea [18].

In addition to the above physiological changes, dry sauna exposure induces significant changes in the composition of the human body. Water is the basic component of the human body (48 ± 6% in females; 58 ± 8% in males) [19]. The body of an average male weighing 75 kg contains approximately 45 liters of water. The turnover rate of water exceeds that of most other body components [20]. Body water is distributed among fluid compartments and different organs [21]. Adequate hydration levels are crucial because the human body copes much better with food shortage than dehydration [20]. Dehydration deteriorates bodily function, including the efficiency of cardiovascular and thermoregulatory systems. Sauna-induced dehydration leads to hyperthermia, which is caused by enhanced sweating [22]. Sweat-induced dehydration, if sufficiently severe, impairs exercise performance and the performance of tasks requiring cognition and skill [23, 24]. Sweat loss amounting to 2% body mass can reduce physical capacity by 20% [25]. BML of 2.5% can lead to a 30% reduction in aerobic power [26]. In women, a decrease in explosive strength caused by rapid, sauna-induced BML was demonstrated by Gutiérrez et al. [27]. Significant dehydration (5% body water loss, BWL) reduces sweating threshold and slope responses [21, 28]. Dehydration greater than 5% BWL may lead to serious health risks, whereas body water loss greater than 8–10% has lethal consequences [29].

Thus, a critical problem in sauna bathing is to minimize dehydration by matching fluid consumption with sweat loss. The extent of dehydration can be estimated by measuring the decrease in body mass [5]. One kilogram of body mass generally corresponds to 1 L of perspired fluid [30, 31]. Thus, measurements of weight loss in a sauna can be useful for estimating the volume of bodily fluids that should be replenished.

In view of the documented risks associated with excessive heat stress in a sauna, BML control is a useful method for counteracting dehydration, hyperthermia, and the related health problems. The question arises as to whether body mass loss (BML) can be reliably estimated based on body mass index (BMI) values in individuals who differ significantly in body dimensions and composition. The existing literature does not provide a comprehensive answer to the above question.

Therefore, the aim of the study was to evaluate the relationship between BMI (independent variable) and BML induced by thermal stress in a dry sauna (dependent variable) in young sedentary women and men.

## 2. Methods

### 2.1. Ethical Approval

The research was carried out upon the prior consent of the Ethical Committee of the University of Warmia and Mazury in Olsztyn. The study was performed on student volunteers who signed an informed consent statement.

### 2.2. Participants

The research was conducted in 2013 on 674 first-year full-time students (326 females, 348 males), aged 19-20 years and enrolled at the UWM. Every volunteer used the sauna during obligatory physical education (PE) classes at the university. The analyzed subjects, both males and females, were residents of villages, towns, and cities (population < 40,000) in the Region of Warmia and Mazury in Poland. The participants attended only mandatory PE classes (90 minutes per week) and did not participate in any other physical activities. The students' physical activity levels were evaluated with the use of the Polish version of the International Physical Activity Questionnaire (IPAQ) [32]. (Neither group fulfilled the “sufficient” criterion because their energy expenditure did not exceed 600 METs/week. Therefore, the subjects were classified as sedentary.) None of the participants had visited a sauna before the study. The following formula [33] was used to determine whether the number of participants constituted a representative sample in the following trial:

(1) $$\begin{array}{c} n=\frac{\mu_{\alpha }^{2}\cdot {\hat{s}}^{2}}{d^{2}}, \end{array}$$

where *d*—maximum (acceptable) error of estimation, *s*—standard deviation, and *μ*_*α*_—value from the normal distribution table *N*(0.1) at the acceptable confidence coefficient 1 − *α* (*μ*_*α*_ = 1.96). At the assumed level of significance, *α* = 0.05, it was presumed that the error of estimation of the average does not exceed 2% [33]. The size of a representative sample, calculated from formula (1) at 2% error of estimation, was 221 females and 198 males, and it was smaller than the studied group (326 women and 348 men). Consequently, the trial can be considered homogenous and representative of UWM students in the 19-20 age group.

### 2.3. Instruments and Procedures

The participants received comprehensive information about the trial before the study. Height was measured to the nearest 0.1 mm and body weight was measured to the nearest 0.1 kg with calibrated WB-150 medical scales with a stadiometer (ZPU Tryb-Wag, Poland). BMI (body mass index, body mass (kg)/body height (m^2^)) was calculated based on somatic parameters. The BMI is widely recognized as an accurate and valid indicator for assessing the degree to which an individual's body mass differs from normal or desirable body weight of an adult with the same height [34]. Deviations from BMI norms were assessed in accordance with the basic WHO classification system [35], and the participants were divided into the following categories:

(2) $$\begin{array}{c} \text{BMI}\leq 18.49\text{—underweight},\\ 18.50\leq \text{BMI}\leq 24.99\text{—normal}\;\text{weight},\\ \text{BMI}\geq 25.00\text{—overweight}\;\text{and}\;\text{obese}. \end{array}$$

The correlations between BMI scores in the three basic classification groups, that is, underweight, normal weight, and overweight and obese, and body mass loss (BML) were determined. BMI was the independent variable (*X*) and BML served as the dependent variable (*Y*). Nude body mass was measured after drying off before and after sauna. The subjects were placed in supine position in a dry sauna (temperature 90°C; humidity 35%) for two sessions of 10 minutes each with a 5-minute break. Immediately after leaving the sauna, the participants cooled for 30 seconds in a paddling pool (pool width—100 cm, pool depth—130 cm, and water temperature—+10°C), and they spent the remainder of the break in a room at a temperature of 25°C and relative humidity of 48%. The subjects were instructed to drink at least 1 L of water on the day before the test and 0.5 L of water 2 hours before the test.

### 2.4. Statistical Analysis

Measurement results were processed in the Statistica PL version 10 application with the use of a descriptive statistics module to calculate the position and distribution of the analyzed factors. Normality of distribution of the analyzed parameters was evaluated in a chi-square test at a significance level of *α* = 0.05 (*P* > 0.05). The significance of differences in BMI values between the percentages of participants in each BMI category, subject to gender, was determined by comparing the two proportions in the *Z*-test. The correlations between BMI and the loss of bodily fluids in the sauna were determined by nonlinear stepwise regression. The results of the regression analysis were presented in a scatter plot [26].

## 3. Results

The number and percentage of students (male and female) in every BMI category are presented in Table 1.

Most women (70.24%) and men (67.53%) were within the normal BMI range; underweight women and men had a 17.18% and 10.34% share of the analyzed group, respectively, whereas overweight and obese women and men accounted for 12.58% and 22.13% of the studied subjects, respectively (Table 1).

The basic anthropometric parameters measured in male and female participants, BMI values, and sauna-induced BML are presented in Table 2.

The data presented in Table 2 points to significant variations in body mass (up to 42.4 kg for women and 70 kg for men) and body height (up to 37 cm for women and 42 cm for men) between the analyzed subjects. The average BMI of both males and females was within the normal range (21.71 kg/m^2^ in women and 23.18 kg/m^2^ in men), whereas the presence of both minimum (16.74 and 17.82, resp.) and maximum (38.41 and 34.46, resp.) BMI values suggests that the analyzed group was highly diverse. The coefficient of variation (CV) for BMI exceeded 15% in women within the range of more than 21 kg/m^2^, and it exceeded 14% in men within the range of more than 16 kg/m^2^ (Table 2).

It was assumed that considerable variations in body mass and BMI values in the evaluated women and men could significantly influence BML induced by thermal stress in the sauna. The analyzed subjects (women and men separately) were divided into three groups based on their BMI scores: underweight, normal weight, and overweight and obese. Sauna-induced BML values were determined for every group (Table 3).

In all three BMI categories, the average BML values were higher in men. The lowest BML was noted in underweight subjects; students with normal body weight lost more weight, whereas the greatest BML was reported in overweight and obese individuals. BML values were particularly high in overweight and obese men and women. BML in overweight and obese women, expressed in terms of percentage BML, was nearly twofold higher than in underweight females. Similar correlations were observed in men.

**Figure 1:**
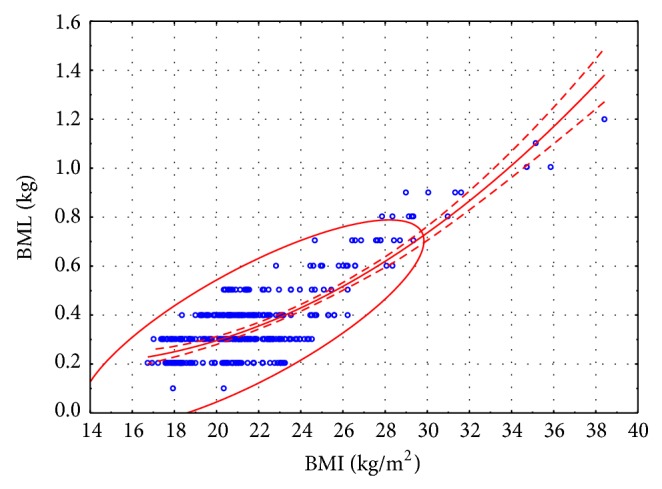
Sauna-induced BML in women from different BMI groups.

**Figure 2:**
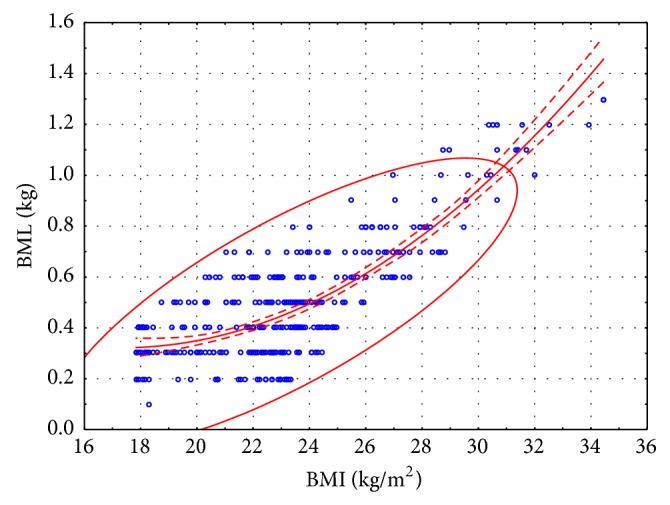
Sauna-induced BML in men from different BMI groups.

The correlations between BMI and BML values are illustrated in Figure 1 (women) and Figure 2 (men). The range of the dataset at confidence level of 0.95 is shown in Figures 1 and 2.

No linear correlations between BMI and BML were reported in female or male subjects.

The following formula (second-degree polynomial) was derived to analyze the dependency between BMI and BML:

(3) For  women:BML=0.4783−0.0445BMI+0.0018BMI2  kg,For  men:BML=1.4803−0.1338BMI+0.0039BMI2  kg.

All of the modeled parameters were statistically significant. The multiple correlation coefficient for the above formula was very high in both sexes at 0.8242 for women and 0.7881 for men.

After stepwise selection, formulas (3) took on the following form:

(4) $$\begin{array}{c} \text{For}\;\text{women:}\;\;\text{BML}=-0.5376+0.0416\text{BMI}\;\;\left(\text{kg}\right),\\ \text{For}\;\text{men:}\;\;\text{BML}=-0.7546+0.0540\text{BMI}\;\;\left(\text{kg}\right). \end{array}$$

In formulas (4) obtained after stepwise selection, the multiple correlation coefficient describing the BML is also high at 0.7988 for women and 0.7770 for men. The correlation between BMI and BML values is directly proportional. A change in BMI by 1 point leads to an average BML change of 42 g in women and 54 g in men.

## 4. Discussion

The main aim of the study was to evaluate the relationship between BMI and BML induced by thermal stress in a dry sauna in young sedentary women and men. It must be noted that for a person with a sedentary to active lifestyle, the average daily water demand is 2–4 L in a temperate climate and 4–10 L in a hot climate [36]. In humans, the most effective method of heat elimination is sweating and evaporation of water from the skin. The inhabitants of desert areas perspire 0.3–1.2 L of water per hour when performing routine daily tasks [25]. Persons performing light exercise in protective clothing generally lose 1.0–2.5 L of water per hour [29]. Individuals consuming a normal diet do not have to drink electrolyte supplements, except for the first hours of exposure to a hot environment [25].

The risks and benefits of sauna bathing have been researched extensively, but most studies were conducted on a small number of participants [13]. During sauna bathing, precipitation is intensified to maintain body temperature at a fairly constant level, which can lead to significant loss of bodily fluids. The magnitude of increase in core temperature ranges from 0.1 to 0.25°C for every percent BML [37]. BML cannot be entirely attributed to dehydration, and it also results from the utilization of energy stores (glycogen, triglycerides) [34]. Both dehydration and overheating can negatively affect basic physiological functions. It should also be noted that the combined consequences of dehydration and hyperthermia are much more severe than the effects induced by dehydration or hyperthermia alone [38].

Fluid replenishment is recommended during and directly after sauna bathing [39]. Incomplete fluid replacement decreases total body water levels [40]. During sauna bathing, sweating begins quickly and reaches its maximum at approximately 15 minutes, with average total secretion of 0.5 kg [15]. The quantity and quality of ingested fluids should be precisely determined. According to experts, sauna-goers should drink 400–800 mL/h of fluids containing 60–80 g of simple sugars and 400–1100 g of sodium during every bathing session [41, 42]. Due to considerable variations in individual sweat rates, it is impossible to design a rehydrating solution that would fully cater to the needs of all sauna-goers [43].

A statistical analysis of the results revealed significant correlations between BMI and BML induced by heat stress in a dry sauna. BML caused by a 20-minute visit to the sauna was determined in the range of 0.24 kg for women with BMI <18.5 and of 0.82 kg for men with BMI >24.99. The noted correlations between BMI and BML values were not linear. Percentage BML in women with BMI >25 was nearly twofold higher than in the group of women with BMI <18.5. Similar correlations were observed in men. The relationship between BMI and BML is directly proportional, which implies that BML increases with a rise in BMI values. The above can probably be attributed to an increase in body surface area, which promotes heat loss through radiation [20]. Our results suggest that persons with a high BMI are more susceptible to dehydration; therefore, they should always replenish fluids during and directly after sauna bathing. The BML values noted in our study are similar to those given by other authors [5, 44, 45].

In a study by Thomas et al. [46], the average BML in 12 healthy adult subjects, induced by a 30-minute visit to the sauna, normalized to body mass was 0.91 ± 0.34% (in the range of 0.33–1.4%). In the work of Coles et al. [47], the BML observed in 10 male subjects (BMI > 27.0) after intermittent sauna bathing sessions (six 15-minute sessions, 48.9°C, with 5-minute breaks) was determined at 0.33 ± 0.19 kg (euhydration trial) and 1.99 ± 0.35 kg (dehydration trial). The change in hydration status was assessed by monitoring changes in urine specific gravity and changes in pretrial body mass. No fluids were ingested at any time during dehydration. The euhydration procedure was identical to dehydration, but during every 5-minute break, the subjects were instructed to drink water in an amount equivalent to the body mass lost during the previous 15-minute sauna session. Euhydration resulted in 0.4% BML, whereas dehydration led to 2.3% BML. Westerterp-Plantenga et al. [48] demonstrated significant changes (*P* < 0.001) in the BML (−1.82 kg; in the range of −1.53 to −2.04 kg; 3.0 ± 0.5%) of 30 nonobese (BMI = 22.8 ± 1.6) and obese (BMI = 28.5 ± 1.9) subjects before and after sauna (three 20-minute sessions, 80°C).

The results of our study and the findings of other authors indicate that sauna bathing has a significant effect on BML. BML is determined by the severity of heat stress (temperature, humidity, exposure time, breaks between sessions, and quantity and quality of ingested fluids), anthropometric parameters, and proportions (BMI). Significant variations were observed between the analyzed individuals, including those between subjects with similar BMI. They can probably be attributed to individual differences in susceptibility to thermal stress (differences in sensitivity and acclimatization to high temperatures), physical endurance, and hydration status. Regardless of the above factors, BML can be estimated based on BMI values. The estimation of BML values has practical significance because it supports the determination of fluid intake required to maintain the body's water balance.

## 5. Conclusions

The results of our study revealed significant correlations between BMI and BML values. BMI is a good predictor of the amount of fluid lost from the body in a dry sauna. An increase in BMI was accompanied by a disproportionate increase in BML, expressed as a percentage of total body mass. Our results indicate that persons with a high BMI are more susceptible to dehydration; therefore, they should always replenish fluids during sauna bathing. The proposed equations for calculating BML values based on a person's BMI can be useful for estimating the amount of fluids that should be replenished by men and women during a visit to a dry sauna. Considerable differences in BML values between subjects with similar somatic parameters (body height, body mass, and BMI) suggest that other factors (independent of BMI) can influence BML. In addition to BML values, body height, and body mass, future studies should include an analysis of body components to determine the contribution of each body component to BML.

## Figures and Tables

**Table 1:** The number and percentage of students (male and female) in three basic BMI categories.

BMI category	Women		Men		Probability (*P*)
	*N*	[%]	*N*	[%]	
Underweight: BMI ≤ 18.49	56	17.18	36	10.34	0.0097
Normal weight: 18.50 ≤ BMI ≤ 24.99	229	70.24	235	67.53	0.4478
Overweight and obese: BMI ≥ 25.00	41	12.58	77	22.13	0.0011

Total	326	100	348	100	—

*Explanations:* BMI: body mass index, *N*: number of respondents, %: percentage, and (*P*): probability of exceeding the calculated value of chi-square.

**Table 2:** Basic somatic parameters and sauna-induced BML in the tested subjects.

Statistical parameters	Somatic parameters, BMI, and BML							
	Women				Men			
	Body mass	Body height	BMI	BML	Body mass	Body height	BMI	BML
	[kg]	[cm]	[kg/m^2^]	[kg]	[kg]	[cm]	[kg/m^2^]	[kg]
Max	85.80	184.0	38.41	1.20	121.00	204.0	34.46	1.3
Min	43.40	147.0	16.74	0.10	51.00	162.0	17.82	0.1
Mean	59.10	164.98	21.71	0.37	75.98	180.73	23.18	0.50
*s*	9.663	7.148	3.293	0.171	13.662	7.528	3.322	0.231
CV [%]	16.35	4.33	15.17	46.97	17.98	4.17	14.33	46.44
Median	56.45	165	21.10	0.30	74.50	180.00	22.96	0.40

*Explanations:* Max: maximum value, Min: minimum value, *s*: standard deviation, CV: coefficient of variation, BMI: body mass index, and BML: body mass loss.

**Table 3:** Sauna-induced BML in three BMI categories comprising women and men.

Statistical parameter	Women						Men					
	Underweight		Normal weight		Overweight and obese		Underweight		Normal weight		Overweight and obese	
	BM	BML	BM	BML	BM	BML	BM	BML	BM	BML	BM	BML
	[kg]	[kg]	[kg]	[kg]	[kg]	[kg]	[kg]	[kg]	[kg]	[kg]	[kg]	[kg]
Max	57.7	0.4	80	0.7	85.8	1.2	68.8	0.4	99.9	0.8	121.0	1.3
Min	43.4	0.1	45	0.1	71.4	0.4	51.0	0.1	51.6	0.2	71.0	0.5
Mean	50.49	0.25	57.93	0.34	77.42	0.70	57.80	0.32	73.08	0.42	93.35	0.82
*s*	3.249	0.056	6.869	0.102	4.533	0.189	3.707	0.080	8.177	0.140	12.304	0.207
CV [%]	6.44	22.86	11.86	30.45	5.86	27.08	6.41	25.24	11.19	33.33	13.18	25.34
BML [%]	0.485		0.578		0.902		0.548		0.575		0.875	

*Explanations:* Max: maximum value, Min: minimum value, *s*: standard deviation, CV: coefficient of variation, BMI: body mass index, and BML: body mass loss.

## References

[1] Hannuksela M. L., Ellahham S. (2001). Benefits and risks of sauna bathing. *The American Journal of Medicine*.

[2] Kauppinen K. (1997). Facts and fables about sauna. *Annals of the New York Academy of Sciences*.

[3] Leppaluoto J., Tuominen M., Vaananen A., Karpakka J., Vuori J. (1986). Some cardiovascular and metabolic effects of repeated sauna bathing. *Acta Physiologica Scandinavica*.

[4] Tatar P., Vigaš M., Jurčovičová J., Ježová D., Štrec V., Palát M. (1986). Impared glucose utilization in man during acute exposure to environmental heat. *Endokrinológia Experimentálna*.

[5] Pilch W., Szyguła Z., Żuchowska M., Gawinek M. (2003). The influence of sauna training on the hormonal system of young women. *Journal of Human Kinetics*.

[6] Prystupa T., Wołyńska A., Ślężyński J. (2009). The effects of finish sauna on hemodynamics of the circulatory system in men and women. *Journal of Human Kinetics*.

[7] Scoon G. S. M., Hopkins W. G., Mayhew S., Cotter J. D. (2007). Effect of post-exercise sauna bathing on the endurance performance of competitive male runners. *Journal of Science and Medicine in Sport*.

[8] Sutkowy P., Woźniak A., Boraczyński T., Mila-Kierzenkowska C., Boraczyński M. (2014). The effect of a single Finnish sauna bath after aerobic exercise on the oxidative status in healthy men. *Scandinavian Journal of Clinical & Laboratory Investigation*.

[9] Podstawski R. (2009). The level of flexibility of the 1st year students of University of Warmia & Mazury in Olsztyn in 2005/2006. *Physical Activity of People at Different Age*.

[10] Tyka A., Żuchowicz A., Kubica R., Pałka T. (2000). Effect of ambient temperature on mechanical power at anaerobic threshold. *Medicine and Science in Sports and Exercise*.

[11] Tyka A., Pałka T., Tyka A. K., Szyguła Z., Cisoń T. (2008). Repeated sauna bathing effects on men’s capacity to prolonged exercise-heat performance. *Medicina Sportiva*.

[12] Ghods M., Corterier C., Zindel K., Kiene M., Rudolf K., Steen M. (2008). Hot air sauna burns. *Burns*.

[13] Kukkonen-Harjula K., Kauppinen K. (2006). Health effects and risks of sauna bathing. *International Journal of Circumpolar Health*.

[14] Papp A. (2002). Sauna-related burns: a review of 154 cases treated in Kuopio University Hospital Burn Center 1994–2000. *Burns*.

[15] Kauppinen K. (1989). Sauna, shower, and ice water immersion. Physiological responses to brief exposures to heat, cool, and cold. Part III. Body temperatures. *Arctic Medical Research*.

[16] Kenefick R. W., Maresh C. M., Armstrong L. E., Riebe D., Echegaray M. E., Castellani J. W. (2007). Rehydration with fluid of varying tonicities: effects on fluid regulatory hormones and exercise performance in the heat. *Journal of Applied Physiology*.

[17] Kukkonen-Harjula K., Oja P., Laustiola K. (1989). Haemodynamic and hormonal responses to heat exposure in a Finnish sauna bath. *European Journal of Applied Physiology*.

[18] Shirreffs S. M., Sawka M. N., Stone M. (2006). Water and electrolyte needs for football training and match-play. *Journal of Sports Sciences*.

[19] Watson P. E., Watson I. D., Batt R. D. (1980). Total body water volumes for adult males and females estimated from simple anthropometric measurements. *The American Journal of Clinical Nutrition*.

[20] Maughan R. J. (2003). Impact of mild dehydration on wellness and on exercise performance. *European Journal of Clinical Nutrition*.

[21] Sawka M. N., Young A. J., Francesconi P., Muza S. R., Pandolf K. B. (1985). Thermoregulatory and blood responses during exercise at graded hypohydration levels. *Journal of Applied Physiology*.

[22] Ylikahri R., Heikkonen E., Suokas A. (1988). The sauna and alcohol. *Annals of Clinical Research*.

[23] Maughan R. J., Shirreffs S. M., Ozgünen K. T. (2010). Living, training and playing in the heat: challenges to the football player and strategies for coping with environmental extremes. *Scandinavian Journal of Medicine and Science in Sports*.

[24] Nybo L., Jensen T., Nielsen B., González-Alonso J. (2001). Effects of marked hyperthermia with and without dehydration on VO2 kinetics during intense exercise. *Journal of Applied Physiology*.

[25] Sawka M. N., Montain S. J. (2000). Fluid and electrolyte supplementation for exercise heat stress. *The American Journal of Clinical Nutrition*.

[26] Luszniewicz A., Słaby T. *Statistics of Computer Package STATISTICA PL. Theory and Applications*.

[27] Gutiérrez A., Mesa J. L. M., Ruiz J. R., Chirosa L. J., Castillo M. J. (2003). Sauna-induced rapid weight loss decreases explosive power in women but not in men. *International Journal of Sports Medicine*.

[28] Montain S. J., Ely M. R., Cheuvront S. N. (2007). Marathon performance in thermally stressing conditions. *Sports Medicine*.

[29] Saltin B., Costill D. L., Horton E. S., Tenjung R. J. (1988). Fluid and electrolyte balance during prolonged exercise. *Exercise Nutrition and Metabolism*.

[30] Maughan R. J., Shirreffs S. M., Leiper J. B. (2007). Errors in the estimation of hydration status from changes in body mass. *Journal of Sports Sciences*.

[31] Sawka M. N., Pandolf K. B., Gisolfi C. V., Lamb D. R. (1990). Effects of body water loss on physiological function and exercise performance. *Perspectives in Exercise Science and Sports Medicine, Volume 3: Fluid Homeostasis during Exercise*.

[32] Biernat E., Stupnicki R., Gajewski A. K. (2007). Międzynarodowy Kwestionariusz Aktywności Fizycznej (IPAQ)—wersja polska. *Wychowanie Fizyczne i Sport*.

[33] Nowak E. (2002). *Outline of Econometric Methods*.

[34] Cole T. J., Bellizzi M. C., Flegal K. M., Dietz W. H. (2000). Establishing a standard definition for child overweight and obesity worldwide: International survey. *British Medical Journal*.

[35] World Health Organization (1995). Physical status: the use and interpretation of anthropometry. *Report of a WHO Expert Committee*.

[36] Brouns F. (1991). Heat—sweat—dehydration—rehydration: a praxis oriented approach. *Journal of Sports Sciences*.

[37] Montain S. J., Coyle E. F. (1992). Influence of graded dehydration on hyperthermia and cardiovascular drift during exercise. *Journal of Applied Physiology*.

[38] Barr S. I. (1999). Effects of dehydration on exercise performance. *Canadian Journal of Applied Physiology*.

[39] Ahonen E., Nousiainen U. (1988). The sauna and body fluid balance. *Annals of Clinical Research*.

[40] Durkot M. J., Martinez O., Brooks-McQuade D., Francesconi R. (1986). Simultaneous determination of fluid shifts during thermal stress in a small-animal model. *Journal of Applied Physiology*.

[41] Kovacs E. M. R., Brouns F. (1997). Nutritonal and physiological aspects of exercise—induced dehydration and rehydration. *Medicina Sportiva*.

[42] Brouns F., Brouns F. (1993). Aspects of dehydration and rehydration in sports. *Nutritional Needs of Athletes*.

[43] Rehrer N. J., Brouns F., Beckers E. J., Saris W. H. M. (1994). The influence of beverage composition and gastrointestinal function on fluid and nutrient availability during exercise. *Scandinavian Journal of Medicine Science and Sports*.

[44] Hawkins C. (1987). The sauna: killer or healer?. *British Medical Journal*.

[45] Kauppinen K., Vuori I. (1986). Man in the sauna. Review article. *Annals of Clinical Research*.

[46] Thomas B. J., Cornish B. H., Ward L. C., Jacobs A. (1999). Bioimpedance: is it a predictor of true water volume?. *Annals of the New York Academy of Sciences*.

[47] Coles M. G., Hernández A., Anderson T. R. (2006). Effect of thermal-induced dehydration on ${\dot{V}\text{O}}_{2}$ recovery time and heart rate response. *Journal of Exercise Science & Fitness*.

[48] Westerterp-Plantenga M. S., Verwegen C. R. T., Ijedema M. J. W., Wijckmans N. E. G., Saris W. H. M. (1997). Acute effects of exercise or sauna on appetite in obese and nonobese men. *Physiology and Behavior*.

